# Cardiac stalling: Modelling energy balance in aortic stenosis and its role in syncope

**DOI:** 10.1113/JP289772

**Published:** 2025-09-19

**Authors:** DeWayne Townsend

**Affiliations:** ^1^ Department of Integrative Biology and Physiology, Medical School University of Minnesota Minneapolis MN USA

**Keywords:** aortic stenosis, coronary flow, *in silico* modelling, syncope

Unimpeded blood flow through the aortic valve is essential for efficient maintenance of cardiac output and the narrowing of this passageway is a common cause of heart disease. Severe aortic stenosis (AS) greatly increases the work of the left ventricle; thus, cardiac hypertrophy and congestive heart failure are not unexpected. However, the increased incidence of syncope and sudden death is difficult to explain (Pellikka et al., 2005). Electrocardiographic data shows that 70% of AS‐associated syncopal events display bradycardia and alterations of the ST segment (Prejean et al., 2021). The sinus bradycardia leading to asystole coupled with hypotension is reminiscent of a reflex arc induced by veratrum alkaloids, the Bezold–Jarisch reflex (BJR). The BJR is characterized by a profound peripheral vasodilatation and a maladaptive bradycardia that arises from conflicting signals from baroreceptors in arterial walls and those in the ventricular wall.

In AS, there is a large pressure drop between the ventricle and the peripheral arteries. This leads to discordance in signals from baroreceptors in the ventricular walls and those of the arteries. Under normal conditions, the signals from these baroreceptors are tightly coupled, with both increasing their activity as pressures rise. Increasing baroreceptor signals inhibit the sympathetic nervous system and activate the parasympathetic nervous system. In the context of AS, the activity of ventricular baroreceptors is higher than that of arterial baroreceptors. This imbalance in baroreceptor activity raises the potential for unchecked cardio depressive actions where hypotensive inactivity of arterial baroreceptors is overwhelmed by hypertensive impulses from the ventricular baroreceptors, resulting in systemic vasodilatation and bradycardia. It is notable that several instances of syncope in patients with AS are proceeded by mild to moderate physical activity, which increase vasodilatation and increases ventricular workload (Prejean et al., 2021). Most AS patients who experience syncope have a history of prior syncopal events (Prejean et al., 2021), suggesting that this inappropriate regulation of cardiac activity is relatively common. Life‐threatening arrhythmic events present a significant prognostic variable and a better understanding of the relative risk of these events have unique clinical relevance.

In this issue of *The Journal of Physiology*, a model is presented by Dvoulety & Sitina (2026) that has the potential to improve prognostic decision making for patients with AS. The model takes a thermodynamic approach based on the assumption that a positive energy balance is required for maintaining the mechanical work performed by the heart and that a negative energy balance leads to cardiac arrest. This model incorporates variables defining cardiac perfusion in the calculation of cardiac energy delivery, including perfusion pressure gradient, oxygen extraction and coronary vascular tone. Meanwhile, the work performed by the left ventricle is defined by the volume of blood pumped and the pressure required to push that blood into the aorta. This latter component is significantly impacted by abnormalities present in AS including the aortic valve diameter, stroke volume, heart rate, ventriculo‐arterial pressure gradients and vascular factors, driven by total peripheral resistance and arterial compliance. These cardiac and vascular variables provide a detailed assessment of the energy provided to the heart by the circulation and the work performed by the myocardium to maintain circulation.

Multivariate analyses of the model reveal that preserving coronary blood flow is the most significant determinant in maintaining a positive energy balance in AS. Surprisingly, the impact of aortic diameter is relatively minor until the severity of the stenosis is severe (i.e. <0.8 cm^2^). In severe AS, the coronary dilatation remains important, but aortic valve diameter, total peripheral resistance and ventricular dilatation also factor into maintaining cardiac function. The importance of aortic valve diameter in the survival of patients with severe AS has been recognized (Pellikka et al., 2005), but the role of peripheral and coronary vasomotor function has not been thoroughly explored in AS related syncope. The frequent presence of ST segment deviation prior to syncope/sudden death highlights the potential role of myocardial ischaemia (Prejean et al., 2021).

Despite the promise of this *in silico* approach, there are several limitations in this model. First, the model assumes a limited maximal coronary vasodilatation potential in AS patients. This assumption is based on an attenuation of coronary dilatation in response to mild ischaemia (Paolisso et al., 2022). It is unclear whether this attenuated response is a limitation of the coronary vasculature or a consequence of left ventricular hypertrophy. Regardless, extension of this coronary dysfunction to instances of severe ischaemia is potentially problematic because data are lacking on the impact of AS on metabolic drivers of coronary vasodilatation that are activated during pathological ischaemia (Duncker & Bache, 2008). Furthermore, the model assumes that oxygen extraction does not change from 80%; however, oxygen extraction does increase as oxygen consumption outstrips oxygen delivery. Thus, the model underestimates energy delivery to the heart during periods of haemodynamic stress. Another limitation of this cardio‐centric model is that the interplay between arterial and ventricular baroreceptors responsible for the acute hypotensive state is largely ignored. How the brainstem responds to the withdrawal of stimuli from arterial baroreceptors defines the severity of the haemodynamic crisis; a rapid activation of the sympathetic nervous system could prevent the syncopal event. Conversely, an exaggerated response to the ventricular baroreceptors could exacerbates the hypotensive episode.

There is a clinical need for the development of prognostic indicators of syncope/sudden death in patients with AS. Detailed haemodynamic models offer a potential means to further personalize risk assessment. Of course, such a model would need to be rigorously validated in AS patients, but models such as that described by Dvoulety & Sitina (2026) may provide a foundation for this work.

## Additional information

### Competing interests

No competing interests declared.

### Author contributions

D.T. was responsible for the conception or design of the work; drafting the work or revising it critically for important intellectual content; and approving of the final version submiited for publication. He agrees to be accountable for all aspects of the work.

### Funding

No funding was received for this work.

## Supporting information


Peer Review History
