# Supplementary material for: Cardiac stalling: Modelling energy balance in aortic stenosis and its role in syncope
Source: J Physiol. 2025 Sep 19;604(13):5082–3. doi: 10.1113/JP289772 (PMC13327753; doi:10.1113/JP289772)
Supplement: Supplementary file 1 — Peer Review History [file TJP-604-5082-s001.pdf]

The Journal of Physiology

Cardiac stalling – Modeling energy balance in aortic stenosis and its role in syncope.

DeWayne Townsend
DOI: 10.1113/JP289772

Corresponding author(s): DeWayne Townsend (town0045@umn.edu)

The following individual(s) involved in review of this submission have agreed to reveal their identity: Michal Sitina (Referee #1)

Review Timeline:

Submission Date:	19-Aug-2025
Editorial Decision:	26-Aug-2025
Revision Received:	29-Aug-2025
Accepted:	04-Sep-2025

Senior Editor: Bjorn Knollmann

Reviewing Editor: Bjorn Knollmann

Transaction Report:

(Note: With the exception of the correction of typographical or spelling errors that could be a source of ambiguity, letters and reports are not edited. Depending on transfer agreements, referee reports obtained elsewhere may or may not be included in this compilation. Referee reports are anonymous unless the Referee chooses to sign their reports.)

Dear Dr Townsend,

Re: JP-P-2025-289772 "Cardiac stalling - Modeling energy balance in aortic stenosis and its role in syncope." by DeWayne Townsend

Thank you for submitting your manuscript to The Journal of Physiology. It has been assessed by a Reviewing Editor and by 1 expert referee and we are pleased to tell you that it is acceptable for publication following satisfactory revision.

Please advise your co-authors of this decision as soon as possible.

The review comments are copied at the end of this email.

Please address all the points raised and incorporate all requested revisions or explain in your Response to Referees why a change has not been made. We hope you will find the comments helpful and that you will be able to return your revised manuscript within 2 weeks. If you require longer than this, please contact journal staff: jp@physoc.org.

Your revised manuscript should be submitted online using the link in your Author Tasks Link Not Available. This link is accessible via your account as Corresponding Author; it is not available to your co-authors. If this presents a problem, please contact journal staff (jp@physoc.org). Image files from the previous version are retained on the system. Please ensure you replace or remove any files that are being revised.

If you do not wish to submit a revised version of your manuscript, you must inform our journal staff (jp@physoc.org) or reply to this email to request withdrawal. Please note that a manuscript must be formally withdrawn from the peer review process at one journal before it may be submitted to another journal.

TRANSPARENT PEER REVIEW POLICY: To improve the transparency of its peer review process The Journal of Physiology publishes online, as supporting information, the peer review history of all articles accepted for publication. Readers will have access to decision letters, including Editors' comments and referee reports, for each version of the manuscript, as well as any author responses to peer review comments. Referees can decide whether or not they wish to be named on the peer review history document.

LANGUAGE EDITING AND SUPPORT FOR PUBLICATION: If you would like help with English language editing, or other article preparation support, Wiley Editing Services offers expert help, including English Language Editing, as well as translation, manuscript formatting, and figure formatting at www.wileyauthors.com/eeo/preparation. You can also find resources for Preparing Your Article for general guidance about writing and preparing your manuscript at www.wileyauthors.com/eeo/prepresources.

REVISION CHECKLIST:

Upload a full Response to Referees file. To create your 'Response to Referees' copy all the reports, including any comments from the Senior and Reviewing Editors, into a Microsoft Word, or similar, file and respond to each point, using font or background colour to distinguish comments and responses and upload as the required file type.

Please upload two versions of your manuscript text: one with all relevant changes highlighted and one clean version with no changes tracked. The manuscript file should include all tables and figure legends, but each figure/graph should be uploaded as separate, high-resolution files. The journal is now integrated with Wiley's Image Checking service. For further details, see: https://www.wiley.com/en-us/network/publishing/research-publishing/trending-stories/upholding-image-integrity-wileys-image-screening-service

You may also upload:
- 'Potential Cover Art' for consideration as the issue's cover image
- Appropriate Supporting Information (Video, audio or data set: see https://jp.msubmit.net/cgi-bin/main.plex?form_type=display_requirements#supp).

We look forward to receiving your revised submission.

If you have any queries, please reply to this email and we will be pleased to advise.

Yours sincerely,

Bjorn Knollmann
Senior Editor
The Journal of Physiology

EDITOR COMMENTS

Reviewing Editor:

Thank you for your Perspective paper. After careful review, I support the minimal suggestions made by the reviewer, which were described in detail, as well as the noted typographical error. Please address these points as part of the final steps towards acceptance.

Senior Editor:

Please consider the request by the reviewer and edit the manuscript if needed.

REFEREE COMMENTS

Referee #1:

We would like to express our sincere thanks for the very interesting Perspectives article on our article!

We have no substantive objections to the piece. We would only like to comment on the statement on line 67: "Regardless, this assumption fails to account for metabolic drivers of coronary regulation that are evident during pathological ischemia (Duncker & Bache, 2008)." In our article, we do not explicitly model autoregulation or vasoreactivity of the coronary vasculature, primarily because we do not have sufficient relevant measured data on coronary resistance to parameterize such mechanisms with adequate precision. Instead, in our simulations we assume that, at the critical instant, autoregulation is maximally engaged; consequently, coronary resistance is set to its minimally attainable value. This minimal value is higher, for example, in the presence of concomitant coronary artery disease. Thus, any regulation, including metabolic control, is implicitly subsumed within our modeling framework.

Additionally, there is a typographical error on line 40: the author's name should read Dvoulety, not Dvoutley.

END OF COMMENTS

Thank you for your appreciative comments, it is interesting work and I'm happy to help highlight it.

Regarding your comments on coronary flow and its regulation. My original version was arguably a little too harsh as the coronary regulation is modeled to the extent that the data is available. The point I was hoping to highlight is that there are types of coronary regulation for which we don't have data on how aortic stenosis impacts them. The data supporting attenuated coronary vasodilation in aortic stenosis comes from mild bouts of ischemia. There are significant data indicating that the vasodilation following mild ischemia is mediated primarily by endothelial cells. However, with severe ischemia other mechanisms working directly on the vascular smooth muscle become more important. There is no data of the impact of aortic stenosis on the coronary vasodilation in response to severe ischemia. The revised version attempts to clarify the distinction between these two forms of coronary regulation and the lack of data from severe ischemia that may alter the response to drops in coronary perfusion pressure.

I have also corrected the error in spelling the author's name.

1st Revision - Editorial Decision 04-Sep-2025

Dear Dr Townsend,

Re: JP-P-2025-289772R1 "Cardiac stalling - Modeling energy balance in aortic stenosis and its role in syncope." by DeWayne Townsend

We are pleased to tell you that your paper has been accepted for publication in The Journal of Physiology.

The last Word (or similar) version of the manuscript provided will be used by the Production Editor to prepare your proof. When this is ready you will receive an email containing a link to Wiley's Online Proofing System. The proof should be thoroughly checked and corrected as promptly as possible.

Authors should note that it is too late at this point to offer corrections prior to proofreading. Major corrections at proof stage, such as changes to figures, will be referred to the Editors for approval before they can be incorporated. Only minor changes, such as to style and consistency, should be made at proof stage. Changes that need to be made after proof stage will usually require a formal correction notice.
All queries at proof stage should be sent to: TJP@wiley.com.

Following copyediting and typesetting the accepted version will be published online.

Yours sincerely,

Bjorn Knollmann
Senior Editor
The Journal of Physiology

IMPORTANT POINTS TO NOTE FOLLOWING YOUR PAPER'S ACCEPTANCE:
If you would like to receive our 'Research Roundup', a monthly newsletter highlighting the cutting-edge research published in The Physiological Society's family of journals (The Journal of Physiology, Experimental Physiology, Physiological Reports, The Journal of Nutritional Physiology, and The Journal of Precision Medicine: Health and Disease), please click this link, fill in your name and email address and select 'Research Roundup':
https://www.physoc.org/journals-and-media/membernews

• TRANSPARENT PEER REVIEW POLICY: To improve the transparency of its peer review process, The Journal of Physiology publishes online as supporting information the peer review history of all articles accepted for publication. Readers will have access to decision letters, including Editors' comments and referee reports, for each version of the manuscript as well as any author responses to peer review comments. Referees can decide whether or not they wish to be named on the peer review history document.

• You can help your research get the attention it deserves! Check out Wiley's free Promotion Guide for best-practice recommendations for promoting your work at: www.wileyauthors.com/eeo/guide. You can learn more about Wiley Editing Services which offers professional video, design, and writing services to create shareable video abstracts, infographics, conference posters, lay summaries, and research news stories for your research at: www.wileyauthors.com/eeo/promotion.

• IMPORTANT NOTICE ABOUT OPEN ACCESS: To assist authors whose funding agencies mandate public access to published research findings sooner than 12 months after publication, The Journal of Physiology allows authors to pay an Open Access (OA) fee to have their papers made freely available immediately on publication.

The Corresponding Author will receive an email from Wiley with details on how to register or log-in to Wiley Authors Services where you will be able to place an order

• You can check if your funder or institution has a Wiley Open Access Account here: https://authorservices.wiley.com/author-resources/Journal-Authors/licensing-and-open-access/open-access/author-compliance-tool.html.

EDITOR COMMENTS

The manuscript is now acceptable. Thank your for your excellent contribution which will be valuable to the field!
